# Bird-Borne Video-Cameras Show That Seabird Movement Patterns Relate to Previously Unrevealed Proximate Environment, Not Prey

**DOI:** 10.1371/journal.pone.0088424

**Published:** 2014-02-11

**Authors:** Yann Tremblay, Andréa Thiebault, Ralf Mullers, Pierre Pistorius

**Affiliations:** 1 Centre de Recherche Halieutique Méditerrannéenne et Tropicale, Institut pour la Recherche et le Développement, Unité Mixte de Recherche 212: IRD-IFREMER-UM2 : Expoited Marine Ecosystems, Sète, France; 2 Percy FitzPatrick Institute and DST/NRF Centre of Excellence, University of Cape Town, Cape Town, South Africa; 3 DST/NRF Centre of Excellence at the Percy FitzPatrick Institute for African Ornithology, Department of Zoology, Nelson Mandela Metropolitan University, South Campus, Port Elizabeth, South Africa; Hawaii Pacific University, United States of America

## Abstract

The study of ecological and behavioral processes has been revolutionized in the last two decades with the rapid development of biologging-science. Recently, using image-capturing devices, some pilot studies demonstrated the potential of understanding marine vertebrate movement patterns in relation to their proximate, as opposed to remote sensed environmental contexts. Here, using miniaturized video cameras and GPS tracking recorders simultaneously, we show for the first time that information on the immediate visual surroundings of a foraging seabird, the Cape gannet, is fundamental in understanding the origins of its movement patterns. We found that movement patterns were related to specific stimuli which were mostly other predators such as gannets, dolphins or fishing boats. Contrary to a widely accepted idea, our data suggest that foraging seabirds are not directly looking for prey. Instead, they search for indicators of the presence of prey, the latter being targeted at the very last moment and at a very small scale. We demonstrate that movement patterns of foraging seabirds can be heavily driven by processes unobservable with conventional methodology. Except perhaps for large scale processes, local-enhancement seems to be the only ruling mechanism; this has profounds implications for ecosystem-based management of marine areas.

## Introduction

Movement ecology [1] has gained impetus in recent years [2], largely as a result of advances in tracking and analytical methodologies. However, our inability to observe an animal's proximate environment at a spatio-temporal scale that approaches the animal's perceptive ability has been a fundamental limitation in the field of movement ecology. This “perceptual sphere” has remained a “black box”, preventing us from accurately linking behavioral processes to observed movement patterns [2]. Consequently, the processes underlying movement patterns are largely unknown and speculative so that the interpretation of distributional data is usually based on assumptions that are not robust.

In recent years, the advent of animal-borne image-capturing devices has provided an opportunity to observe what happens within the visual field of an animal [3]. A number of pilot studies revealed interesting behavioral aspects of foraging animals such as intra or inter species interactions [4]–[6], foraging substrate selection [7] and the use of tools [8], but none has linked video observations with high resolution movement records (but see [9] for an example with 1 minute time-lapse photography). In the absence of real observations, animals are often assumed to move in relation to a number of suspected important parameters, such as prey distribution or other environmental conditions.

The Cape gannet (*Morus capensis*) makes a useful model to explore the importance of external stimuli in governing foraging behaviour in seabirds. These birds congregate in large numbers during the breeding season at six islands along the southern African coast, where they are readily accessible for deployment of instrumentation. They primarily feed on spatially dynamic shoaling epipelagic fish species such as sardines (*Sardinops sagax*) and anchovy (*Engraulis encrasicolus*) [10], [11] and we have a poor understanding on how these food resources are located. Cape gannets do form foraging flocks at sea, involving other seabirds and marine mammals [12]. In order to form these associations, Cape gannets must move in relation to these other predators after they have encountered prey. Similarly, northern gannets affect their route with respect to the presence of fishing boats [9]. Hence, we hypothesized that movement patterns should change with respect to the various sources of information (i.e. cues or stimuli) available to the foragers (such as dolphins, boats, seabirds...). Further, these cues being different in number, displacement speed, distribution and visibility, we suggest that movement patterns must carry characteristics specifics to the cues.

In the present study, we used data recorded with miniaturized video-cameras and GPS recorders fitted to breeding Cape gannets in order to understand which stimuli might drive their movement patterns while foraging and how movement patterns relates to external stimuli.

## Materials and Methods

This study was carried out in strict accordance with the recommendations from the South-African National Parks – SANParks. Procedures used in this study are standards and non-invasives. Steps taken to ameliorate animal suffering include 1) Light equipment 2) Attachment of equipment on plumage using removable tape 3) Only one individual of the pair was used for study to limit impact on chicks 4) Short deployment period (only one trip at sea recorded, 1-2 days). The protocols have been specifically approved by the SANParks Animal Use and Care Committee (AUCC), and access to the island was permitted by SANParks.

Fieldwork took place at Bird Island (33° 50' 26.6'' S, 26° 17' 14.5'' E), Algoa Bay, South Africa, between the 7^th^ and 28^th^ of December 2010. We fitted 36 chick-rearing Cape gannets with a micro-video-camera (Camsports nano, Camsports™, Estrablin, France) and a GPS (i-GotU GT-600, Mobile-Action-Technology Inc., Taipei, Taiwan) in order to record fine-scale foraging behavior of this threatened species. The video camera (68 mm length, 19 mm diameter, 22 grams) provided us with 736×480 pixels images at 25 frames per seconds (fps) with a 74° lens angle, for about 1.5 hours. The GPS units were reconditioned from their original packages and cast in epoxy resin for shock and water resistance. They measured 43×40×12 mm and weighed 36 g after modification and were set to record GPS location every 5 seconds allowing for the full foraging trip to be recorded. The two loggers were consolidated as a single unit using a custom-made thin Kevlar strip (∼2 g) which was attached to the lower dorsal feathers with adhesive tape (Tesa™, Hamburg, Germany). The whole package weighed 70–75 g, corresponding to ∼2–3% of the birds' body mass. The units were recovered after a single foraging trip. The information from the video records was extracted and merged with positional following [13].

In order to relate a particular movement pattern with a given foraging context, we first determined the foraging context for each observed dive. Three foraging contexts were observed: a dive was considered “with dolphins”, “with fishing boat” or “with other gannets” if it occurred within 5 min of the respective observations on the video. Second, because most of the descriptors of movement such as speed or sinuosity are dependent on the scale at which they are measured, and because marine foragers operate at several embedded scales [14], we measured movement metrics at three distinct scales. As there is no consensus on how should the scale be measured (i.e. distance, area, circle radius etc.?) [15] we used segments of the track, the length of which defines the scale. Centered on each dive, we selected a segment that measured two, six and twelve kilometers in total length. These three scales were chosen empirically based on careful observations of the tracks. Segments of two kilometers typically encompass localized foraging patterns at the diving site, such as a succession of loops. Segments of six kilometers encompass medium scale movements that could be described as “hopping between potential diving sites”. Segments of 12 km are long enough to describe relocations from a foraging area to another one. It is important to note that these values are adapted to our dataset and cannot be generalized. In order to test the sensitivity of these values on the final results, we replicated the analysis using some variations in these numbers (1,5, 10 km, and 2,8, 20 km). The results were virtually the same (data not shown). In each segment, we measured the average speed, the standard deviation of the speed, the sinuosity (total traveled distance/straight distance between the beginning and the end of the segment), and the fractal dimension, as an index of space-coverage index, following [15].

We used a decision tree as a classification procedure in order to relate movement descriptors (response data) with foraging context (nominal predictor data). We used a bagging procedure [16] with 200 iterations in order to better estimate the classification errors. The process consists in generating 200 trees based on a random subset of the data (the bag). The out-of bag data were then used to estimate the out-of-bag error, a reliable estimate of the ensemble error [16].

The wind data were derived from the Cross-Calibrated Multi-Platform Ocean Surface Wind Vector L3.0 First-Look Analyses:

http://podaac.jpl.nasa.gov/dataset/CCMP_MEASURES_ATLAS_L4_OW_L3_0_WIND_VECTORS_FLK. Documentation can be found at:

"ftp://podaac-ftp.jpl.nasa.gov/allData/ccmp/L3.0/docs/ccmp_users_guide.pdf". Wind data were available at 6 hours intervals. The wind data was chosen in time and space to correspond to the position of the birds after 1 hour at sea (2 thirds of the data collection period). The wind data associated with each of the three foraging situations were stable in time, and the data obtained were likely to have been a true reflection of foraging conditions.

Data analysis was performed using Matlab (R2010a, The MathWorks, Natick, MA, USA).

This study was carried out in strict accordance with the recommendations from the South-African National Parks (SANParks).

## Results

**Figure 1:**
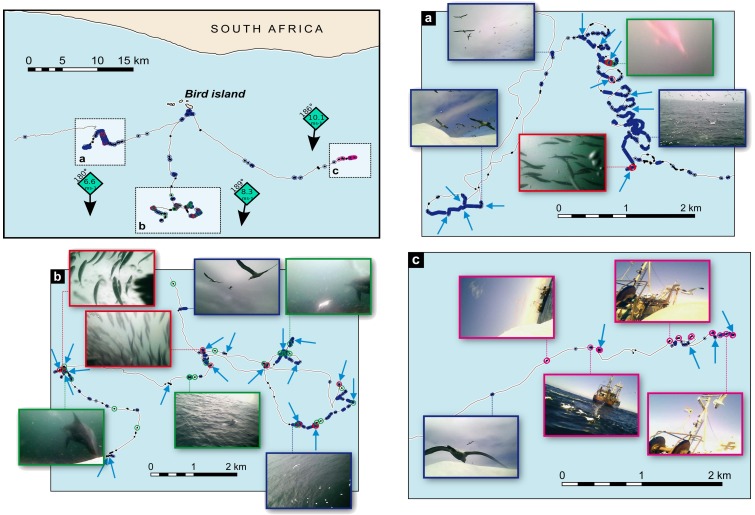
Portions of GPS tracks and video-camera images recorded concurrently, in Cape Gannets. Dots represent events seen in the images. For each track, the wind speed and direction (clockwise from true North) is given. **a) b) c)** Zoom in each of the tracks. Arrows indicate dives, blue = other gannets, red = prey, green = dolphins, magenta = boat.

Out of the 35 deployments that provided usable data (additional table S1), particularly sinuous movement patterns (visually determined, Figure 1) were recorded in 10 birds (always in the context of group foraging) and only three birds seemed to have foraged alone during the camera running-time. All others had joined other predators, con-specific or not. Therefore, over 90% of birds were associated with other predators. Despite the fact that only the first 1.5 hours (∼10%) of the foraging trips were recorded (additional table S1), diving was observed from 14 instrument-carrying birds, with gannet, dolphin and trawler-associated feeding occurred in 79%, 36% and 7% of these birds, respectively. Diving was generally not associated with directional movement. However, images showed that during these relatively straight-trajectories the birds reacted to the presence of other birds, by joining them and/or orientating in an opposite direction. Given the challenge of quantifying coupled video and GPS data, we chose to qualitatively describe three out of the 35 records in order to exemplify how combined video and tracking data allows for an understanding of stimuli that drive the birds' trajectories (Figure 1). These three records were selected to represent all foraging situations observed.

### The beginning of foraging trips

Foraging trips of the three birds began in a similar fashion. Shortly after take-off, they landed on the water near the colony with other gannets and preened. They then flew in the direction from which other birds were returning to the colony, suggesting information transfer between those birds. During out-going flights, they formed small groups (often 3–5 individuals). Landings, reorientations in relation to other birds, and group flights were repeated several times prior to the location of feeding sites.

### Gannet-associated flock feeding, Figure 1a

Fifteen minutes (13 km from the island) after leaving the colony the number and frequency of gannets in the images increased rapidly, as the study bird approached a large feeding flock of >150 gannets. The bird initiated its first dive 1–2 minutes after arriving at the feeding aggregation. The bird then engaged in a series of nine ∼0.5 km diameter clockwise loops that spread over two kilometers. During the 18 minutes it took to do this, a total of seven dives were performed. Prey, other gannets and dolphins were occasionally visible, showing that this feeding aggregation was multi-specific. Loops were not superimposed suggesting that the school of fish had moved straight in response to predation, at an estimated speed of 6.7 km per hour. Our study bird then relocated ∼3 km away in a South-Westerly direction, joining other gannets flying in the same direction, and reached another flock of >80 foraging gannets where it dived four times before stopping on the water surface for ∼11 minutes. The bird then returned to where it left the first school of fish 25 minutes earlier but the flock was gone. During this time, the school of fish could not have been further than 2.8 km away (assuming speed estimated previously). The bird did not search for the school but instead, initiated another South-Westerly flight, apparently alone. The camera stopped recording during this flight. The GPS however continued sampling positions and the data indicated a relatively straight trajectory of ∼15 km followed by a high level of tortuousness in its movement. The initial directional movement suggests that the bird aimed at this specific locality. The camera data showed that the coastline was clearly visible at ∼17 km distance, but the resolution was too low to distinguish other gannet at this distance.

### Dolphin-associated feeding, Figure 1b

The first dive of this study bird took place at ∼16 km from the colony, in association with other gannets. No dense flocks of gannets were observed, but instead, birds were far apart (estimated 50–100 m). The highest densities of birds (groups up to 80 birds) were found during feeding bouts. The bird flew from site to site, with other birds often seen flying in the same direction, with short periods of time spent at each site, even after diving. During 40 minutes, the bird dived 18 times at 13 different sites (>500 m from each other) spread longitudinally within a 7×3 km zone. Within this area, 20 dolphins could be seen. The faster movement of gannets in relation to dolphins suggests that most of the dolphin sightings were different individuals or groups. Other Cape gannets were always visible around dolphins. The track pattern resulting within this ecological context was a medium scale (500 m–7 km) tortuousness, with sudden changes in azimuth followed by directional movements, occasionally interrupted by small scale (∼50 m) zig-zags or loops usually at the diving sites.

### Trawler scavenging, Figure 1c

This study bird rapidly approached a fishing boat and dived next to it. The boat was a South-African trawler in fishing operation (flag and cables were visible at the back) at about 26 km East South-East from Bird Island. A group of ∼20 gannets and at least one kelp gull (*Larus dominicanus*) were also present. The bird dived four times around the boat before the camera stopped recording. After each dive, the bird stayed for relatively long periods of time (1 to 6+ minutes) drifting on the water before taking off again, and this seemed to be the case for other gannets around the boat. As gannets move much faster than trawlers they probably adopt a sit-and-wait strategy, rather than circling the vessel. Consequently, the overall movement pattern was relatively straight and often interrupted with pauses, zig-zags and loops as the bird regains close contact with the boat and dive around it. Given the initial directional movement during an extended time period, it seems clear that the bird was heading towards the boat at a distance of seven kilometers (although the boat was not visible on the images from this distance). Later during the rest of the trip (GPS data only), other similar patterns of movement suggested that the bird encountered other fishing boats.

### Classifying the ecological context around dives locations

**Figure 2:**
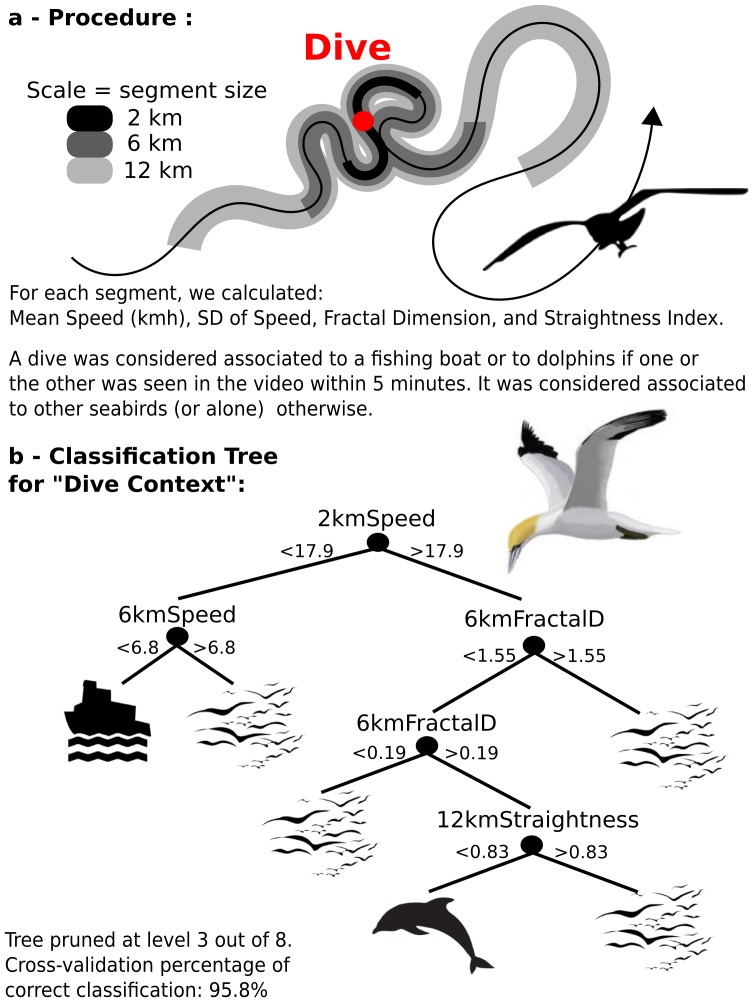
Determining the foraging context using GPS data only, in Cape Gannets. Procedure a) and results b) of the classification-tree classifying the foraging context around diving events using some track characteristics.

These descriptions of foraging activity can be inferred from the trajectories' characteristics, using data from all of the 35 birds. Given the numerous pauses on the water surface during boat-foraging periods, the dives performed around boats can be flagged by low flying speeds calculated both at two and six km scales (Figure 2). Foraging in association with dolphins involve different patterns, and are best described by high speed at a 2 km (small) scale, intermediate fractal dimension at 6 km (medium) scale and a relatively low straightness index at 12 km (large) scale (Figure 2). Interestingly the best classifiers involved several scales and the fractal dimension (over 6 km segments) is the second most important factor. The variations (SD) in speed did not appear to be discriminant. Cross-validation for the pruned-tree in figure 2 showed a high percentage of correct classification (95.8%). However, the more robust estimate of correct classification obtained using the bagging procedure indicate a cumulative out-of-bag error of around 0.12, indicative of a lower – but still high – correct classification percentage (88%).

Applying these classification rules to the portions of tracks for which we do not have video data to infer the foraging context (i.e. ∼90% of the tracks) revealed that 100%, 90.9% and 90.9% of birds foraged at least once in association with other gannets, with dolphins, and with fishing boats, respectively. However, the average (± SD) occurrence of these association was 33.3 (± 30.0), 5.5 (± 5.2) and 19.9 (±33.0) times per trips for gannet, dolphin, and fishing boat associated feeding, respectively (n = 35).

## Discussion

The detailed description of combined video and GPS data has proven extremely powerful in terms of understanding movement patterns, because it provided an immediate understanding of the movement context and causation. However, the quantification of such processes is challenging and would require further methodological developments. On another hand, the simple description of video events and the associated animal movement patterns highlights and facilitates an understanding of processes in an obvious and compelling manner. Except for the three birds that seemed to have been alone for the time of the video recording, all others showed systematic movement adjustments in relation to other foragers, showing a remarkably high prevalence of information sharing in these birds. The three patterns described above correspond to known aspects of the ecology of these birds [10], [12], however it was enlightening to see how patterns of movement were typical of given ecological contexts.

Predators within the proximate environment of Cape gannets were the main drivers of their movement patterns at scales roughly < 20 km. It is unlikely that foraging bird relied on olfactory senses as the wind directions were such that the use of smell would not have aided prey patch location (Figure 1). Foraging was furthermore exclusively diurnal, demonstrating the reliance on visual hunting. Given the obvious superiority of the bird's eye compared to the camera, and the high contrast of the birds on the seascape, it seems possible that our study birds visually cued on other foraging birds up to 15–17 km, as previously suggested for other seabirds species [17]. Gannets generally fly in clockwise loops when many other gannets are present around the breeding colony before landing at their nests (pers. obs.). As collisions rarely happen, but can be fatal [18], looping pattern could represent an anti-collision strategy, and may suggest the presence of a high density of aerial predators.

With video data available for only about 10% of the foraging trips, we were limited in associating movement patterns to the respective foraging contexts. It is important to note that the first 10% of these tracks were mostly associated with locating foraging zones and the beginning of foraging phases in some cases. From a methodological standpoint, we propose that 90 minutes of camera autonomy is required to enable foraging phases to be recorded for Cape gannets and other similar study species. The results from the extrapolation of the foraging contexts from the first 10% to the remaining 90% of the trip using the classification tree need to be interpreted carefully, because the foraging phases were determined as short low-speed periods, without certainty about dive occurrence. The results might therefore be overestimated. However, the number of putative dives averaged 62 per trip, which is very similar to what has previously been reported at the same breeding period for this species (66 and 68 dives per trip at Lambert's bay and Malgas respectively [19]). Despite this uncertainty, our extrapolation thus appears plausible and confirms the very high prevalence of the use of other predators in shaping the movement patterns in these birds during the whole trip. Interestingly, a similar proportion of birds might have used boats and dolphins during their trips, but the actual estimated occurrences suggest that boats were much more often used as compared to dolphins. This is possibly due to the fact that boats are more visible than dolphins. It therefore appears that boats form a strong attraction to these seabirds, thus potentially impacting time budgets and foraging efficiencies. Interestingly, we did not find fishery discards in the diet of Cape gannets during the study period (data not shown) suggesting that boats are used as cues for inferring the presence of live prey. The high prevalence of boat association that we estimated for Cape gannets in Algoa bay is very similar to recent findings in northern gannets *Morus bassanus* [9], although the later study did not mention dolphins associations, possibly as a result of lower sampling frequency of images (1 min) which might limit the probability of detecting elusive underwater predators.

The high prevalence of the use of other foragers in shaping the movement patterns in Cape gannets shows that local enhancement mediated through information transfer is fundamental in determining foraging decisions. A recent modeling study showed that informed movement processes are much more efficient than those that are non-informed [20]. As a result, the number of behavioral changes in a track increases when other predators are present [13]Our study takes it one step further and show that informed movements are the rule, and that information mostly comes from the predatory guild. This has profound implications because then, the predator community structure is of primary importance and a prey-rich environment alone might not suffice for these animals. When considering the paramount importance of facilitation between these predators, it becomes reasonable to argue that the relative effects of competition could be negligible, except under conditions of extreme prey shortage.

Our data provide a mechanistic explanation for discrepancies observed between seabird and prey distributions or remote-sensed oceanographic variables [21], [22]. Local enhancement induces local peaks in predator densities leaving other zones free of predators thus deviating from the hypothesis of an ideal-free distribution [23]. Our results further explain why gannets are not necessarily associated with clearer-waters were prey are more visible [24] and show that seabirds do not look for prey, but instead, it is likely that their cognitive search image is oriented towards cues of the presence of prey (mostly other predators). This distinction is fundamental if we are to understand foraging flexibility and adaptation to change. Reasonable changes in the amount of prey might not be as important as changes in the predator community structure. As such, changes in the fishing fleet, dolphins, sharks, seals, other seabirds or whale populations and their relative distributions might considerably affect the foraging efficiency of seabirds and their ability to cope with changes in prey availability. This also brings to light the importance of the at-sea community of juveniles and non-breeding con-specifics. Those individuals are not as constrained as breeding seabirds to commute to the colony, and their permanent presence at sea might allow for the maintenance of a functional foraging network enabling some elements of the population to remain in proximity of prey while others commute to the breeding sites.

Finally, our results suggest that research focused on the quantification of interactions within the predatory guild and on the sensory abilities of predators, are necessary in order to gain further insights on the likelihood that these species will be able to successfully adapt to a rapidly changing world.

## Supporting Information

Table S1 **Main characteristics of the data collected.**
(PDF)
