# Supplementary material for: Bird-Borne Video-Cameras Show That Seabird Movement Patterns Relate to Previously Unrevealed Proximate Environment, Not Prey
Source: PLoS One. 2014 Feb 11;9(2):e88424. doi: 10.1371/journal.pone.0088424 (PMC3921161; doi:10.1371/journal.pone.0088424)
Supplement: Table S1 — Main characteristics of the data collected. (PDF) [file pone.0088424.s001.pdf]

ID	Date	GPS Record (h)	GPS record complete ?	Video Record (h)	% of GPS record with video	Other Predators Seen ?	Seen During Transit	Seen during diving phases	Multispecific association when diving ?	Dives observed ?
CAGA_001	08-Dec-2010	21.32	No	1.49	-	Gannets	Gannets	Gannets	No	Yes
CAGA_002	10-Dec-2010	15.55	Yes	1.42	9.12	Gannets	Gannets	Gannets	No	Yes
CAGA_003	10-Dec-2010	22.52	No	0.53	-	Gannets	Gannets	n.a.	n.a.	No
CAGA_004	10-Dec-2010	1.52	No	1.31	-	Gannets, Dolphins	Gannets, Dolphins	Gannets	No	Yes
CAGA_005	11-Dec-2010	7.68	Yes	1.44	18.78	Gannets	Gannets	-	No	Yes
CAGA_006	11-Dec-2010	19.78	Yes	1.47	7.45	Gannets	Gannets	n.a.	n.a.	No
CAGA_007	12-Dec-2010	5.44	Yes	1.34	24.68	Gannets, Dolphins, Cormorants, Storm Petrel	Gannets	Gannet, Dolphins, Cormorants, Storm Petrel	Yes	Yes
CAGA_008	12-Dec-2010	18.32	Yes	1.44	7.85	Gannets	Gannets	n.a.	n.a.	No
CAGA_009	13-Dec-2010	8.03	Yes	0.33	4.10	Gannets, Gulls	Gannets, Gulls	n.a.	n.a.	No
CAGA_010	13-Dec-2010	22.74	Yes	1.48	6.49	Gannets	Gannets	n.a.	n.a.	No
CAGA_011	13-Dec-2010	24.67	Yes	1.14	4.63	Gannets	Gannets	n.a.	n.a.	No
CAGA_012	13-Dec-2010	21.49	Yes	0.95	4.41	Gannets	Gannets	n.a.	n.a.	No
CAGA_013	14-Dec-2010	4.62	Yes	1.40	30.34	Gannets	Gannets	n.a.	n.a.	No
CAGA_014	15-Dec-2010	6.28	Yes	1.40	22.36	Gannets	Gannets	n.a.	n.a.	No
CAGA_015	15-Dec-2010	21.52	Yes	1.39	6.45	Gannets	Gannets	Gannets	No	Yes
CAGA_016	15-Dec-2010	24.79	No	1.44	-	Gannets, Cormorants, Penguin	Gannets	Gannets, Cormorants, Penguin	Yes	Yes
CAGA_017	14-Dec-2010	19.59	No	1.51	-	Gannets	Gannets	n.a.	n.a.	No
CAGA_018	17-Dec-2010	6.66	Yes	1.45	21.73	Gannets, Dolphins	Gannets	Gannets, Dolphins	Yes	Yes
CAGA_019	17-Dec-2010	21.04	Yes	1.30	6.17	Gannets	Gannets	n.a.	n.a.	No
CAGA_020	17-Dec-2010	24.26	Yes	1.48	6.10	Gannets, Fishing Boat, Cormorants, Gulls	Gannets	Gannets, Fishing Boat, Cormorants, Gulls	Yes	Yes
CAGA_021	18-Dec-2010	21.44	Yes	1.40	6.51	Gannets, Dolphins, Cormorants	Gannets	Gannets, Dolphins, Cormorants	Yes	Yes
CAGA_022	14-Dec-2010	16.84	No	0.85	-	Gannets	Gannets	n.a.	n.a.	No
CAGA_023	19-Dec-2010	8.00	Yes	0.86	10.79	Gannets, Penguins	Gannets, Penguins	n.a.	n.a.	No
CAGA_024	19-Dec-2010	17.07	Yes	1.41	8.29	Gannets, Dolphins, Gulls	Gannets	Gannets, Dolphins, Gulls	Yes	Yes
CAGA_025	19-Dec-2010	22.10	No	1.34	-	Gannets	Gannets	n.a.	n.a.	No
CAGA_026	20-Dec-2010	21.60	No	0.85	-	Gannets	Gannets	Gannets	No	Yes
CAGA_027	21-Dec-2010	23.14	Yes	1.44	6.23	Gannets	Gannets	Gannets	No	Yes
CAGA_028	21-Dec-2010	-	-	-	-	-	-	-	-	-
CAGA_029	21-Dec-2010	22.23	No	1.48	-	Gannets, Dolphins	Gannets	Gannets, Dolphins	Yes	Yes
CAGA_030	21-Dec-2010	25.04	No	1.46	-	Gannets	Gannets	n.a.	n.a.	No
CAGA_031	23-Dec-2010	20.87	Yes	1.41	6.75	Gannets, Dolphins, Cormorants, Storm Petrel	Gannets	Gannets, Dolphins, Cormorants, Storm Petrel	Yes	Yes
CAGA_032	23-Dec-2010	21.86	No	1.49	-	Gannets	Gannets	n.a.	n.a.	No
CAGA_033	24-Dec-2010	16.42	Yes	0.11	0.69	-	-	-	-	-
CAGA_034	25-Dec-2010	7.65	Yes	1.46	19.10	Gannets	Gannets	-	No	Yes
CAGA_035	14-Jan-2011	25.29	No	0.55	-	Gannets	Gannets	n.a.	n.a.	No
CAGA_036	14-Jan-2011	28.02	No	1.44	-	Gannets	Gannets	n.a.	n.a.	No

Additional Table 1 : Main characteristics of the data collected.
n.a. Stands for “not aplicable”.
Note that Penguins, Cormorants, Storm Petrels and Gulls are all anecdotical observations. They are observed in very little numbers and for a very short time. Their determination at the species level was not always possible. Penguins were surely African Penguins (*Spheniscus demersus*), Cormorants were dark-breasted cormorants, most likely Cape cormorants (*Phalacrocorax capensis*). Storm petrels were unidentified. Dolphins were most likely long-beaked common dolphins (*Delphinus capensis*), but indo-pacific bottlenose dolphins (*Tursiops aduncus*) could also be present. Gulls were most likely kelp gull (*Larus dominicanus*) in all instances. The sex of our study birds is unknown.
